# Towards Therapeutic Applications of Arthropod Venom K^+^-Channel Blockers in CNS Neurologic Diseases Involving Memory Acquisition and Storage

**DOI:** 10.1155/2012/756358

**Published:** 2012-06-04

**Authors:** Christiano D. C. Gati, Márcia R. Mortari, Elisabeth F. Schwartz

**Affiliations:** ^1^Departamento de Ciências Fisiológicas, Instituto de Ciências Biológicas, Universidade de Brasília, 70910-900 Brasília, DF, Brazil; ^2^Universidade Católica de Brasília, 71966-700 Brasília, DF, Brazil

## Abstract

Potassium channels are the most heterogeneous and widely distributed group of ion channels and play important functions in all cells, in both normal and pathological mechanisms, including learning and memory processes. Being fundamental for many diverse physiological processes, K^+^-channels are recognized as potential therapeutic targets in the treatment of several Central Nervous System (CNS) diseases, such as multiple sclerosis, Parkinson's and Alzheimer's diseases, schizophrenia, HIV-1-associated dementia, and epilepsy. Blockers of these channels are therefore potential candidates for the symptomatic treatment of these neuropathies, through their neurological effects. Venomous animals have evolved a wide set of toxins for prey capture and defense. These compounds, mainly peptides, act on various pharmacological targets, making them an innumerable source of ligands for answering experimental paradigms, as well as for therapeutic application. This paper provides an overview of CNS K^+^-channels involved in memory acquisition and storage and aims at evaluating the use of highly selective K^+^-channel blockers derived from arthropod venoms as potential therapeutic agents for CNS diseases involving learning and memory mechanisms.

## 1. Introduction

Many efforts have been made to understand the physiological mechanisms responsible for learning and memory. Due to their complexity, different approaches have been used to unlock them and various actors of these phenomena have been often revealed [1, 2]. In the last two decades, a new agent has gained the attention of the scientific community studying the processes of learning and memory: the potassium channels [3]. 

Potassium channels (KCNs) exhibit a great diversity (for review see [4, 5]). In mammals, nine and ten genes that encode channels for Na^+^ and Ca^2+^ have been described, respectively. Nonetheless, for KCN they are 78 genes, at least [5]. In addition to this large number of genes, alternative splicing, RNA editing, posttranslational modifications, and channel formation of heteromeric assembly by the association of different principal subunits also contribute to the diversity of KCN [4]. These channels can be grouped into four families: voltage-gated channels (K*_v_*), calcium-activated channels (K_Ca_), inward-rectifiers channels (K_ir_) and two tandem-pore channels (K_2P_). Furthermore, these families have many subfamilies each containing several members: K_*v*_ have 12 subfamilies (K*_ v_*1-K*_ v_*12), K_Ca_, 5 subfamilies (K_Ca_1-K_Ca_5), the K_ir_, 7 subfamilies (K_ir_1-K_ir_7), and K_2P_, 15 subfamilies (K_2P_1-K_2P_7, K_2P_9,K_2P_10, K_2P_12, K_2P_13 and K_2P_15-K_2P_18) [6–9].

**Figure 1:**
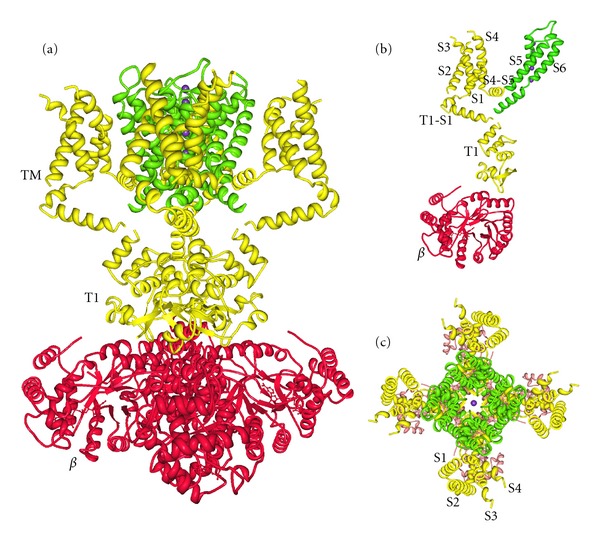
Views of the K_v_1.2-*β*_2_ subunit complex. (a) Side view of the K_v_1.2-*β*_2_ structure with the extracellular solution above and the intracellular solution below. Four subunits of the channel are colored in yellow (T1 domain and voltage sensor) and green (pore). *β* subunit tetramer is colored in red. TM indicates the integral membrane component of the complex. (b) Stereoview of a single subunit of the channel and *β* subunit viewed from the side. Labels correspond to six transmembrane helices (S1 to S6). (c) View of the K_v_1.2-*β*_2_ structure from the extracellular side of the membrane. S1–S4 helices from each subunit form four surrounding voltage-sensing domains, S5-S6 regions (green) from the four subunits shape a single pore domain. Purple spheres are potassium ions. Images were generated using Protein Workshop Viewer 3.9 [15] and Protein Data Bank accession ID 2A79 [14].

The four families of KCNs are structurally related. K_v_, K_ir_, and K_Ca_ are transmembrane proteins formed by four *α*-subunits, whereas in K_2P_ there are only two. *α*-Subunits of all K_v_, K_Ca_2, and K_Ca_3 have six transmembrane segments (6TM), while in K_Ca_1, K_Ca_4, and K_Ca_5 are 7TM. All K_ir_ have *α*-subunits with 2TM and those of K_2P_ contain 4TM. When *α*-subunits join to form a channel, they may be identical and thus generating homodimeric or homotetrameric (homomultimeric) channels or may be different resulting in heterodimeric or heterotetrameric (heteromultimeric) channels. Heteromultimeric channels are formed by the association of different *α*-subunits of the same subfamily. *α*-Subunits assembly of K_2P_ forms two pores and the in other KCNs there is one pore (for reviews, see [4, 5, 10–13]). The X-ray structure of mammalian K_v_1.2 channel with the *β*_2_ subunit (Figure 1) was reported by Long et al. [14]. In accordance with what is mentioned above, K_v_ channels are formed by four *α*-subunits that generate one pore (Figures 1(a) and 1(c)). Each K_v_1.2 channel subunit contains six transmembrane segments, termed S1 to S6 (Figure 1(b)). The S5-S6 regions (Figures 1(a)–1(c), green) from the four subunits shape a single pore domain. The S1-S4 helices from each subunit form four surrounding voltage-sensing domains (Figures 1(b) and 1(c)).

KCNs have a significant influence on the neuron's activity, functioning of neuronal circuits, and brain plasticity. These channels regulate action potential firing patterns and control neurotransmitter release by constraining local membrane excitability and limiting Ca^2+^ influx (for review, see [16]). Furthermore, KCNs participate in the induction of synaptic plasticity by shape excitatory postsynaptic potentials (EPSPs) and enhance synaptic integration through their N-methyl-D-aspartate receptor (NMDAR), the cellular analogue for learning and memory (for review, see [3]). Therefore, KCNs may have an important role in cognition.

This possible involvement of KCN in cognitive processes is reinforced by its strong presence in Central Nervous System (CNS). These channels have a wide distribution in the brain of mammals. Several members of the K_v_ families [17–20], K_Ca_ [21–25], K_ir_ [26–29], and K_2P_ [30, 31], are found in the telencephalon, diencephalon, brainstem, and cerebellum of mammals.

The use of KCN blockers has aided to elucidate the function of these channels in the CNS. The tetraethylammonium (TEA) and 4-aminopyridine (4-AP) are traditional (classical) pharmacological blockers for KCN [32]. However, they are not specific, neither act on all types of KCN. TEA blocks different subtypes of K_v_ channels (except K_v_4.2, K_v_4.3, K_v_5.1, K_v_7.1 and all of the subfamilies K_v_6, K_v_8-K_v_12), K_Ca_ (except subfamily *K*_Ca_2 and K_Ca_3), and subtypes K_ir_3.4 and K_ir_7.1, but has no effect on K_2P_ channels (for review see [6–9]). The 4-AP also operates on different subtypes of K_v_ channels (except K_v_3.4, K_v_5.1, and all K_v_6-K_v_12), K_ir_3.4, and K_ir_7.1, but not on K_Ca_ channels, neither on the K_2P_ [6–9]. Therefore, the potential of these blockers to investigate the role of KCN on cognitive processes is limited.

Their diversity, distribution, and function suggest that the potassium channels could be involved in different cognitive processes, leading to a complex scenario that embarrass the understanding of the role of each K^+^-channel subtype in these events. This challenge is even more complicated by the lack of drugs that act specifically on each type of KCN. One way to solve this problem has been the use of toxins isolated from invertebrates' venoms as K^+^-channels blockers.

Many animal toxins, such as those isolated from spider, scorpion, and bee venoms, are specific blockers of potassium channels. While TEA operates in millimolar concentrations, scorpion toxins bind to and block K_v_ channels with pico- or nanomolar affinities [33]. Apamin, a toxin isolated from the bee venom, blocks K_Ca_2.2 and K_Ca_2.3 channels with affinity of the order of picomolar [34]. Therefore, animal venom toxins may be more useful as pharmacological tools than the classical blockers (TEA and 4-AP) as they act on K^+^-channels with high potency and selectivity [35].

The aim of this paper is to review the knowledge accumulated on the importance of KCN to the processes of learning and memory. In addition, we present the contribution and potential use of toxins isolated from spiders, scorpions, and bees as pharmacological tools in this investigation. Finally, the participation of KCN in clinical conditions holding cognitive deficits and the possible use of toxins from animals as therapeutic agents are also considered.

## 2. Potassium Channels in Learning and Memory

The hippocampus has a great importance in learning and memory processes. This limbic structure holds a key in the consolidation of explicit memory. It receives information of events and transfers them to the neocortex where they are stored for a period (even weeks) and then gradually returned to specific regions of the cerebral cortex contributing to the formation of long-term memory [36].

The hippocampus expresses many types of KCN. As shown in Table 1, 39 different types of KCN belonging to the four families (K_v_, K_Ca_, K_2P_, and K_ir_) have been identified in this neural structure. Of these, the more expressed channels in the hippocampus appear to be the K_v_7.2, K_v_7.3, K_Ca_1.1, K_ir_3.2, and K_ir_3.3, followed by K_v_1.1, K_v_1.6, K_v_3.1, K_v_4.2, K_v_10.1, K_Ca_2.1, K_Ca_2.2, and K_ir_3.1 (Table 1). What would be the role of this KCN diversity in the hippocampus?

Many experimental studies (Table 2) show that KCN may have a significant contribution in learning and memory processes. In these studies, the activity or expression of K^+^ channels in the brain of rats and mice was altered by different strategies. The impact of this manipulation on the learning and memory was accessed by behavioral tests.

Ghelardini et al. [50] worked with mice subjected to passive avoidance test (Table 2). They found that intracerebroventricular (i.c.v.) administration of minoxidil, pinacidil, and cromakalim (KCN openers) produced amnesia. However, TEA, gliquidone and glibenclamide (KCN blockers) prevented this effect. These researchers also used toxins (apamin and charybdotoxin) whose results will be addressed later in the present paper.

Vick et al. [53] studied mice submitted to object recognition task, contextual fear-conditioning paradigm, and tone fear-conditioning paradigm (Table 2). Systemic 1-ethyl-2-benzimidazolinone (EBIO) and cyclohexyl-[2-(3,5-dimethylpyrazol-1-yl)-6-methyl-pyrimidin-4-yl]-amine (CyPPA), K_Ca_2 channel activators, impaired the encoding, but not retrieval, of object memory in a spontaneous object recognition task. In addition, EBIO did not affect contextual or cued fear memory. They also tested apamin and this treatment will be discussed in Section 4.1.

Matthews and Disterhoft [54] observed rats submitted to trace eyeblink conditioning (Table 2). Intrahippocampal (CA1) injection of paxilline, a K_Ca_1.1 channel blocker, resulted in slowed learning of the task.

Hammond et al. [55] produced transgenic mice that overexpress K_Ca_2.2 subunits by 10-fold and tested them by Morris water maze, contextual fear-conditioning paradigm, and tone fear-conditioning paradigm on mice (Table 2). They found that this condition impaired learning in all tasks. The same results for these animals in contextual fear-conditioning paradigm were obtained for Stackman et al. [56].

Jacobsen et al. [57] tested doxycycline-induced conditional K_Ca_2.3-deficient mice in five distinct learning and memory paradigms: passive-avoidance test, Morris water maze test, object recognition task, Y-maze test, and five-trial inhibitory avoidance test. Impairment was only observed in the last two tasks (with no effect on the others).

Deng et al. [58] worked with rats subjected to Morris water maze test (Table 2). They infused small interfering RNA (siRNA) in the entorhinal cortex (EC) to knock down K_2p_10.1 channels. Baclofen, a specific *γ*-aminobutyric acid (GABAB) receptor agonist, was also applied into EC. The treatment of rats with siRNA abolished baclofen-induced inhibition of spatial learning. When administered alone, siRNA tended to improve the learning ability of rats.

Wickaman et al. [46] employed mice lacking a functional K_ir_3.4 gene and submitted them to passive-avoidance test and Morris water maze test (Table 2). These mice performed similarly to wild-type controls in the first task, however, exhibited impaired performance in latter.

Betourne et al. [48] worked with wild-type and K_ir_6.2 knockout mice submitted to Morris water maze test, contextual fear-conditioning paradigm, and tone fear-conditioning paradigm (Table 2). In wild-type mice, intra-hippocampal (CA3) injection of diazoxide (K_ir_6.2 opener) impaired contextual memory. This effect was reversed by co-injecting tolbutamide (K_ir_6.2 blocker). The K_ir_6.2 knockout mice presented impairment of contextual and tone memories and slightly impaired performance in Morris water maze (special memory).

It has been described that K_v_1.1 channels contribute to the processes of learning and memory. Meiri et al. [51] were able to inhibit the expression of K_v_1.1 in the hippocampus, blocking the translation of the mRNA of these channels. They found that this procedure worsened the passive avoidance in mice and spatial memory in rats (Table 2). Kourrich et al. [59], working with odor-discrimination tasks in rats, showed that levels of mRNA expression of K_v_1.1 channels in the hippocampus were positively correlated with associative learning.

On the other hand, K_v_2.1 channels appear to interfere negatively with learning and memory. Zhong et al. [40] suggested that memory deficits induced by scopolamine in rats may result from exacerbation of potassium currents in hippocampal pyramidal neurons as a consequence of increased mRNA expression of K_v_2.1 channels.

It has been shown that K_v_12.2 (BEC1) channels have preferential distribution in the forebrain, including the hippocampal and cortical regions [19, 60, 61]. The use of K_v_12.2 knockout and overexpression (OVER) mice made possible to verify that this KCN is negatively involved in cognitive function, since the K_v_12.2 knockout mice performed behavioral tasks related to working memory, reference memory, and attention better than their wild-type animals. In the OVER mice, on the other hand, the performance of those tasks was impaired [52] (Table 2).

Taken together, these studies strongly suggest that the brain KCNs and their modulation play an important role in the regulation of memory processes. Some hippocampal K_v_ (K_v_1, K_v_2 and K_v_12), K_Ca_ (K_Ca_1 e K_Ca_2), and K_ir_ (K_ir_3 e K_ir_6) channels seem to be particularly relevant. On this basis, the KCN blockers could be useful in investigation and the treatment of cognitive deficits.

## 3. Arthropod K^**+**^ Channels Toxins

Arthropod venoms constitute a rich source of peptidyl KCN inhibitors (KTxs). There are two classes of inhibitory peptides based on their mechanism of action: (1) KCN blockers which bind to the outer vestibule and then blocking the ion conductance by the pore occlusion; (2) KCN gating modifiers which shift the channel opening to more positive potentials. Scorpion KCN blockers (scorpions KTxs), exemplified by charybdotoxin (ChTX) [62], and the peptide tertiapin, isolated from bee venom [63], act as pore blockers, while spider KTxs, such as SGTx1 [64], act as a gating modifier. In turn, apamin, another toxin from bee venom, possibly acts as a pore blocker, although residues of the extracellular S3/S4 loop of the K_Ca_2 (SK) channels also affect the apamin binding [65]. According to Lamy et al. [66], based on differences in binding affinity and potency of the blockage, apamin does not behave as a classical pore blocker, and probably the blocking effect occurs by an allosteric mechanism.

These KTxs show different arrangement of their three-dimensional (3D) structures. The folding types earlier found are *αα*, *αββ*, and *β* *αββ* [67–69]. Despite the conformation differences, most of these peptides have common residues which promote the binding with the potassium-channel vestibule, such as a lysine residue distant from an aromatic residue for 6.6 ± 1.0 Å [70].

Arthropod toxins have been used as pharmacological tools to better understand the role of ion channels, as most of them act in a high specific and potent way. Some of these toxins constitute unique blockers of certain ion channels, such as ergtoxin-1 (*Centruroides noxius*) [71], and BeKm-1 (*Mesobuthus eupeus*) [72] for K_v_11.1 (HERG), psalmotoxin (*Psalmopoeus cambridgei*) for ASIC1a channel [73], tertiapin-Q (*Apis mellifera*) for K_ir_3.1 (GIRK) [63], and Lq2 (*Leiurus quinquestriatus hebraeus*) for K_ir_1.1 (ROMK1) [74].

The scorpion KTxs are formed by 20–95 amino acid residues stabilized by two, three, or four disulfide bonds, making this structure relatively stable. The scorpion KTxs were originally classified into three families named *α*, *β*, and *γ* [75], all of them have the highly conserved secondary structural arrangement *α*/*β* stabilized by cysteines (CS*α*/*β*). More recently, scorpion KTxs presenting a different structural arrangement, with only two *α*-helices stabilized by two disulfide bonds, CS*α*/*α*, were described, and these peptides were named *κ*-KTxs [76–78]. Among the almost 190 scorpion KTxs described until now, the *α*-KTx family, the largest one, contains more than 130 peptides thus far, classified in 20 subfamilies, based on their amino acid homology [75, 79].

Toxins from spider venom can play an important and complementary role in investigation of KCN cognitive function. Unlike most animal toxins obtained from snakes, bees, scorpions and sea anemones venom, which block mainly K_v_1 and K_v_3 channels, peptide toxins from spiders target K_v_2 and K_v_4 channels, which are expressed in the CNS and cardiovascular system of mammals (for review see [80]). Moreover, the venom spiders belonging to the Theraphosidae family represent a plentiful source of peptides that modify the gating of K_v_ channels [81]. Hanatoxin and seemingly others tarantula toxins shift channel opening to more depolarized voltages [81, 82] by stabilizing the resting conformation of the voltage sensor [83]. It has been suggested that these peptides interact with the voltage-sensor paddle within the lipid membrane [84–86].

The bee venom is composed of several classes of peptides, as well as enzymes and biogenic amines. Among the peptides, it can be emphasized the presence of the melittin [87], apamin [88], tertiapin [89], and mast cell degranulating peptide [90]. Certainly, in relation to neurotoxins, the apamin has greatly excelled, since this peptide has been considered a good pharmacological tool and it has provided important information in respect of the functioning of K^+^ -channels [91].

## 4. Arthropod KTxs in Learning and Memory

As already mentioned above, KCN classical blockers are not specific and cannot act at all KCNs. So these blockers have a major limitation as tools for studying the role of KCN in the CNS. An alternative to this obstacle is the use of KTxs from arthropods venom. In this section we report studies using these toxins to access the mechanisms of learning and memory in animals submitted to behavioral tests.  Table 3 presents a summary of these works. Analyzing this table, we can see that the apamin is the most used KTx in tasks of learning and memory, as detailed below.

### 4.1. Apamin

Apamin is a small peptide that corresponds to less than 2% of bee venom dry weight. Its amino acid sequence was described independently by two groups, revealing completely its primary structure [104]. Like many other peptide neurotoxins, apamin has high cysteine content and a high basicity, but apamin is different from most peptide toxins in its unusual ability to cross the blood brain barrier and act on the Central Nervous System [105]. This peptide is a polypeptide of 18 amino acids having a molecular weight of 2039 Da, with two disulfide bridges connecting position 1 with 11, and position 3 with 15 [106]. According to Vincent et al. [107], the most important part of the apamin sequence for neurotoxic activity appears to be the C- terminal region containing the two arginine residues, given that chemical modification of Argl3 and Arg14 eliminates toxicity (DL_50_ in mice). Assays with autoradiography of binding sites for apamin revealed that it binds preferentially to the hippocampus, to the habenular nucleus, and to the nucleus medialis septi [108].

 Apamin is an antagonist of all three subtypes of small conductance Ca^2+^-activated K^+^-channel, K_Ca_2 channels. However, this KTx showed subtype-specific affinity demonstrated by values of half maximal inhibition (IC_50_) and, dissociation constant (Kd) for K_Ca_2.1 (SK1), K_Ca_2.2 (SK2), and K_Ca_2.3 (SK3) (IC_50_ = 704pM, 27pM, 4 nM and Kd 390pM, 4pM, 11pM, resp.) [34]. As a consequence of pharmacological blockage, apamin has been considered as a useful tool to investigate the physiological mechanisms involved in higher brain functions, especially cognitive processes or in the control of mood [98, 109].

This bee KTx facilitates spatial and nonspatial learning and improves memory performance in laboratory rodents, accelerating the acquisition of bar-pressing response in appetitively motivated mice [94]. In that report, when apamin was injected (dose 0.2 mg/kg, i.p.) 30 min before the acquisition session, it accelerated lever-press learning and the performance in a retention session 24 h later in BALB/mice. Results showed that immediate administration improved the retention of the lever-press task one day later [94]. Additionally, administration of the apamin in the acquisition of the lever-press task produced a stronger increase in early gene expression, *c-fos* and *c-jus*, in the CA1, CA3, and dentate gyrus as compared to trained saline-injected mice [110]. The similar pattern of immediate genes has been observed in the initial activation of neurons during the memory process [99].

Moreover, Deschaux et al. [95] showed that injection of apamin (0.4 mg/kg i.p.) before the training improved learning in an object recognition task in rats. Emphasizing, they found that rats injected with apamin before the first exploration session spent more time in exploring the new object than the familiar object at the second trial, when it took place 24 h after the first trial. Injection of apamin just after the first trial or before the second trial did not modify the difference in exploration time between the new and the familiar object. These results suggest that apamin could improve learning, but not consolidation or restitution of the information, in an object recognition task [95].

Also in 1997, Deschaux and Bizot [96] reported that, in the habituation task, apamin (0.4 mg/kg, i.p) decreased activity (distance travelled and rearing) on the restitution sessions only when it was injected before acquisition sessions, but not when injection took place just after the acquisition session or before the restitution session. In addition, the authors showed that in the passive avoidance test, apamin did not alter performance whenever the time of administration. According to Deschaux and Bizot [96], blockage of the apamin sensitive K_Ca_ channels improved the acquisition in nonstressful task, but not in a stressful situation in rats.

In the water maze spatial navigation, apamin (0.2 and 0.06 mg/kg, i.p) administered 30 min before daily training improved the acquisition and reversal learning of septal-lesioned mice, but it did not improve learning and memory in a spontaneous alternation task in a Y-maze and in a passive avoidance task, and did not affect learning and memory in any of three tasks when intact mice were used as subjects [97]. Interestingly, apamin dose dependently (0.2–0.06 mg/Kg, i.p) reversed the lesion-induced defect in the radial arm maze and in the water maze [99]. These results corroborated that blockage of K_Ca_ channels can alleviate the spatial reference memory and working memory defect induced by a damaged septohippocampal axis [99].

Van Der Staay et al. [98] make a comprehensive study using series of cognition tests with mice and rats from different strains. They used the standard version and a modified version of the Morris water escape task, the passive and active avoidance tasks, and the operant tasks in the Skinner box as cognitive tests, and the rat forced swimming test and the open-field test after cocaine administration as noncognitive tests. Results showed that apamin appeared to improve the cognitive performance of mice in two versions of the Morris water escape task and the passive avoidance task. However, inconsistence evidence was identified in rats. Moreover, apamin affected the general behavior of rats in the visual discrimination task, in the forced swimming test, and in the open field after cocaine administration [98].

In 2001, it was tested the effect of an i.c.v apamin (0.3 ng) injection on an olfactory associative task. Apamin did not modify the learning of the procedure side of the task or the learning of the odor-reward association. To specifically test reference memory, the rats were trained on a new odor-association problem using the same procedure (acquisition session), and they were tested for memory retention 24 h later. Apamin injected before or after the acquisition session improved retention of the valence of a new odor pair. Thus, the results indicate that the blockage of apamin-sensitive K_Ca_2 channels facilitates reference memory [100].

In order to investigate the effect of potassium channel subtypes on rats learning and memory, Mpari et al. [101] compare the effects of two blockers, apamin (0.3 ng) and lei-Dab7 (3 ng), a modified scorpion KTx that selectively blocks K_Ca_2.2 [111]. The results indicated that the blockage of K_Ca_2.2 and K_Ca_2.3 channels by apamin facilitates consolidation on new odor associations, using olfactory associative task in rats. However, lei-Dab7 remains without effect suggesting an involvement of K_Ca_2.3 channels for the integration of synaptic signaling and plasticity modulation involved in learning and memory processes [101].

In the same way, in a spatial radial-arm maze task with rats, it was shown that lei-Dab7 did not modify attention or memory. However, apamin (specific to K_Ca_2.2 and K_Ca_2.3 channels) improved reference memory and accelerated strategy changes from egocentric to allocentric. These results reinforce that K_Ca_2.3 blockage improves memory in rats [25].

Apamin is also able to facilitate the encoding of contextual fear memory during the limited 1 conditioned stimulus-unconditioned stimulus pairing protocol. It was shown that mice treated with apamin exhibited significantly greater freezing during the context test than did the saline-treated control [53].

More recently, the role of K_Ca_ in memory formation was explored in chicks trained on a single-trial discrimination avoidance task. Blockage of K_Ca_2 channels using apamin (1 nM, 0.02 ng/hem, i.c.) impaired long-term memory retention when administered between 10 min before, and 30 min after training [103].

Blockage of K_Ca_2 channels in the prefrontal cortex (PFC) by apamin also improves working memory performance [102] (Table 3). In prefrontal, visual, and somatosensory cortical pyramidal neurons, K_Ca_2 channels mediate a hyperpolarization following calcium-induced calcium release, triggered by activation of muscarinic [112, 113] or glutamate receptors [114]. The PFC is central for working memory, the aptitude to internally symbolize information without an external input, being fundamental for establishing conceptual thinking, and for language, for example. As K_Ca_2 channels play an important role in diminishing the excitability during synaptic transmission in the medial prefrontal cortex (mPFC), their blockage could potentiate synaptic transmission optimizing activity within the mPFC network [115, 116].

All these studies suggest that K_Ca_2 channels are involved in memory processes but only when the task does not implicate a spatial strategy or a stressful situation. Since apamin is effective in some of these examples, but fails to have an effect in others, it appears that apamin-sensitive channels affect only certain circuitries involved in memory processing. Van Der Staay et al. [98] discuss the use of apamin as a tool to study the role of potassium channels in learning and memory. They do not consider apamin a good tool, despite its high selectivity, because the peptide has a very narrow therapeutic window, since there is an apparent overlap between the doses that enhance cognition with those causing side effects. Even so, studies using apamin are of great importance for the understanding of these channels and their influence on processes of learning and memory.

### 4.2. Charybdotoxin

Charybdotoxin (*α*-KTx 1.1, ChTX-Lq1, or ChTx-a), isolated from *Leiurus quinquestriatus hebraeus* (Yellow scorpion) [117], is a potent selective inhibitor of high (large or big) conductance Ca^2+^-activated potassium channels (K_Ca_1.1, BK, or maxi-K), as well as a K_v_1.3 channel [62]. In an autoradiographic study of rat brain it was demonstrated high levels of [^125^I]-charbydotoxin in white matter regions such as the lateral olfactory tract and fasciculus retroflexus, as well as in gray matter-containing regions such as the zona incerta, medial geniculate, and superior colliculus [118].

Using a [^14^C]-2-deoxyglucose autoradiographic technique, it was shown that i.c.v. administration of charybdotoxin produced effect on glucose utilization in 21 brain regions predominantly limited to the hippocampus, limbic and motor structures, indicating that glucose utilization was altered within three pathways implicated within learning and memory processes, the septohippocampal pathway, Schaffer collaterals within the hippocampus, and the Papez circuit. These results suggested the possibility that handling of particular subtypes of K_v_1 channels by specific scorpion toxins in the hippocampus and related structures could alter cognitive processes without provoking large-scale changes in neural activity throughout the brain [119].

Ghelardini et al. [50] showed that the i.c.v. administration of charybdotoxin, 20 min before the training session in the mouse passive avoidance test, prevented the amnesia induced by potassium-channel openers (minoxidil and pinacidil). The amnesia in mice was produced by opening the K_ATP_ potassium channels, and it was reversed by blocking K_ATP_ potassium channels. Since the prevention of the amnesia induced by K_ATP_ potassium channel openers in the mouse passive-avoidance test is also obtained by blocking voltage-gated and calcium-activated channels, it is plausible to consider that more than one type of KCN appears to be involved in cognitive processes. It is worth mentioning that KCN blockers used by Ghelardini et al. [50] did not improve cognitive abilities when given alone, contrasting with the findings showing that apamin enhances memory in an object recognition task [95]. In fact, apamin, as it blocks K_Ca_ channels, affects learning rather than memory, an effect that is noticeable only in an object recognition task and that was observed after an interval of 24 h, when the control animals were not able to remember the exploration of the objects presented in the first session anymore [50].

### 4.3. Kaliotoxin

Kaliotoxin is a specific inhibitor of K_v_1.1 and K_v_1.3 isolated from the scorpion *Androctonus mauretanicus mauretanicus* [120]. The involvement of K_v_1.1 and K_v_1.3 in learning and memory processes was studied by using kaliotoxin in rats submitted to olfactory associative learning. Kaliotoxin (10 ng) improved learning but not information consolidation in the odor-reward training and increased the long-term retrieval of an odor-reward association tested by a reversal test 1 month after the odor-reward training. The reference memory was also tested by successive odor-pair training. When kaliotoxin was injected before the acquisition or retention session it improved performance. Nevertheless, when kaliotoxin was injected immediately after acquisition no effect was observed, suggesting that the blockage of K_v_1.1 or K_v_1.3 channels by kaliotoxin facilitates cognitive processes as learning, in particular in a reference representation [92].

### 4.4. Iberiotoxin (IbTx)

IbTx, a toxin isolated from venom of the scorpion *Mesobuthus tamulus*, is a selective inhibitor of K_Ca_1.1 channels [121, 122]. These channels have been identified in brain regions that are related to cognition such as the hippocampus [123], and they have also been implicated in memory processing.

In rabbits, it has been shown a strong relationship between classical conditioning and membrane excitability in Purkinje cells which persisted for at least 1 month [124]. In slices of cerebellar lobule, the administration of IbTX mimics the increases in membrane excitability related to conditioning, suggesting that classical conditioning is dependent upon the inhibition of K_Ca_1.1 channels [124].

In chicks submitted to passive avoidance task, Edwards and Rickard [93] showed a transient retention loss (40–70 min after training) associated with the central administration of 50 nM IbTX immediately after training.

### 4.5. Spider KTxs

Despite K_v_2 channels possibly contributing negatively to the mechanisms of learning and memory and spider venom being a rich source of gating modifiers of K_v_2 channels, no evaluation of the use of the spider KTxs as probes in cognitive processes was carried out so far. Only one study [125] tested the effect of a spider toxin in mice submitted to behavioral tests of memory, but this toxin was not a KTx.

## 5. Potassium Channels and Clinical Conditions Holding Cognitive Impairment

The studies presented above suggest that, at least in part, expression modification and conductance modulation of potassium channels are related to learning and memory processes. In some neurological dysfunctions, the relationship between KCN and cognitive aspects is also evident.

### 5.1. Limbic Encephalitis

Limbic encephalitis is characterized by disorientation, agitation, anxiety, depression, irritability, personality change, acute confusional state, hallucinations, complex partial and secondary generalized seizures, and, mainly, by impairment of short-term memory. Limbic encephalitis may have paraneoplastic or autoimmune origin (for reviews see [126, 127]).

Some cases of limbic encephalitis reinforce that KCNs are involved in learning and memory processes. Patients with this clinical condition and high plasma concentration (as reflected in the cerebrospinal fluid) of antibodies against voltage-dependent potassium channels (anti-K_v_) had severe memory impairment. This cognitive function has a neuropsychological improvement after reducing serum levels of anti-K_v_ by plasma exchange, immunosuppressive treatment or spontaneous fall [126, 128–130].

### 5.2. Human-Immunodeficiency-Virus-Type-1 (HIV-1-) Associated Dementia (HAD)

HAD is a severe and debilitating form of HIV-1-associated neurocognitive disorders (HANDs). Over 40 million people worldwide are infected by HIV and 20–30% of them displayed symptoms of HAD. This disorder is characterized by cognitive deficits, motor disturbances, and behavioral abnormalities (for reviews see [131, 132]).

In individuals with HAD, the cognitive deficits could be result of K_v_ channels dysfunction [133, 134]. The brain cortex in individuals with HAD revealed overexpression of genes coding for KCNs that prolong afterhyperpolarization [135].

This participation of KCN in memory in the clinical condition caused by HIV-1 is supported by experimental results. Injection of HIV-1-infected human monocyte-derived macrophages into brain of immunodeficient mice, a model of human HIV-1-encephalitis (HIVE), impaired both long-term potentiation in hippocampus and spatial learning [136, 137]. Interestingly, these two damages are reversed when the HIVE mice received systemic administration of 4-AP, a KCN blocker aforecited [138].

### 5.3. Schizophrenia

Schizophrenia is a neuropsychiatric disorder that affects approximately 0.8% of the world population. It is characterized by the presence of several symptoms that can be grouped into two categories: positive and negative symptoms as a function of normal behavior. Positive symptoms (in addition to normal behavior) include hallucinations, delusions, and disorganized thoughts. Negative signs (absent in normal behavior) consist of anhedonia and social withdrawal [139, 140].

Schizophrenia is associated with cognitive deficits [141]. Weickert et al. [142] found that 51% of 117 patients with schizophrenia and decline in intelligence quotient (IQ) also exhibited deficits of executive function, memory, and attention. Deficits of executive function and memory also were found in 71% of 73 individuals with schizophrenia that showed intellectually compromised or deteriorated [143].

This memory deficit observed in patients with schizophrenia may be also consequence of dysfunction of potassium channels. Early life exposure of rodents to maternal separation or social isolation is an animal model for schizophrenia [144]. Quan et al. [145] observed deficits in learning and memory in postweaning isolation-reared rats similar to individuals with schizophrenia. When compared to the housed rats, the isolated ones performed worse in probe trials and memory retention tests in Morris water maze. Interestingly, these researchers found that the amplitudes of hippocampal voltage-dependent transient potassium A-type (*I*_(A)_) currents were enhanced, and the steady inactivation curve of *I*_(A)_ currents was shifted towards positive potential by CSF of isolated rats. These K^+^ currents regulate action potential backpropagation and the induction of specific forms of synaptic plasticity, which is thought to underlie learning and memory [146, 147]. Therefore, Quan et al. [145] suggest that the mechanism by which early stressful experience leads to long-lasting consequences for spatial memory involves hippocampal potassium ion channel currents.

### 5.4. Alzheimer's Disease

Disorders that impair mental abilities are among the most feared result of aging. Among the kind of dementia, Alzheimer's disease (AD) represents the substantial majority of cases. AD is a progressive neurodegenerative disease characterized by loss of function and death of neurons in various areas of the brain, leading to loss of mental functions such as memory and learning. The clinical diagnosis of AD is usually done when the memory loss speeds up, and other behavioral and cognitive symptoms appear, mainly because the failure in the capacity to remember ordinary facts of everyday life is easily dismissed as normal aging. The episodic memory is related to the hippocampus and the interconnections circuits between this area and cortex goes through changes during aging that are highly susceptible to neurodegeneration in AD. Unfortunately, by the time of AD diagnosis, prominent neuronal loss has already occurred in the entorhinal cortex (EC), the brain's interface between the hippocampal formation and neocortex. It is worthy to mention that neurodegeneration in EC does not occur in healthy brains undergoing aging, and as structural and functional measures of the EC, dentate gyrus and CA3 region circuit display a progressive change on the course to AD. This circuit could represent a suitable target to therapies aimed to modify the disease progression [148].

In addition to the decreased number of acetylcholine (Ach) receptors in the basal forebrain cholinergic neurons (BCFN), K_v_3.1 and K_v_2.1 have been implicated to AD. The use of immunohistochemical techniques showed that both KCNs are expressed in the BCFN [149]. BFCN voltage-gated KCNs regulate Ach release and may participate in BFCN neurodegeneration in the course of AD.

The accumulation of amyloid beta plaques is an AD characteristic. It seems that these plaques form calcium-conducting ion channels that cause rapid neurodegeneration due to calcium overload [150]. Toxins acting as Ca^2+^ channel blockers, such as *ω*-agatoxin, attenuate the increases in Ca^2+^ concentration in isolated hippocampal nerve endings in the rat [149].

*I*_(A)_ currents and the KCN behind these currents seem to have a role in AD. KCNs responsible for *I*_(A)_ currents have been involved in the onset of long-term potentiation in mammalian neurons, which is thought to underlie learning and memory [146, 147]. A change in the steady-state properties of the *I *_(A)_ current was showed in the amyloid-treated *Drosophila* cholinergic neurons which was sufficient to increase the threshold for the initiation of repetitive firing [151]. In this study, specific KTxs of the K_v_4.2 channel (phrixotoxin-2) and K_v_1 channel (*α*-dendrotoxin) were used to determine which channels triggered these currents in the *Drosophila* model. It had been shown that treatment of cholinergic neurons with amyloid peptide altered the kinetics of the current and caused a decrease in neuronal viability [152].

Brain microglia and their KCNs are also important to AD. These cells are activated to produce a “respiratory burst,” which produces reactive oxygen species (ROS) that cause the death of target cells in pathological states [153]. However, ROS generated by microglia contribute to the death of neurons in neurodegenerative conditions such as Alzheimer's and Parkinson's diseases, HIV and prion infection, and multiple sclerosis (reviewed by [154]). K_v_1.3 and K_Ca_2 and K_Ca_4 are required for the respiratory burst and ROS formation in cultured microglia, and the inhibition of K_v_1.3 by agitoxin-2 prevented neuronal killing by the microglia [155]. Moreover, charybdotoxin and *α*-dendrotoxin, all K_v_1.3 blockers, reduced neuron killing by microglia as shown by transwell cell-culture system [156].

### 5.5. Multiple Sclerosis

Multiple sclerosis (abbreviated MS, known as *disseminated sclerosis* or *encephalomyelitis disseminata*) is an inflammatory disease. In MS, the fatty myelin sheaths around the axons of the brain and spinal cord are destroyed, leading to demyelination and scarring as well as a broad spectrum of signs and symptoms such as muscular weakness, loss of coordination and speech, visual disturbances, and cognitive disability. MS is a chronic degenerative disease and the most common neurological cause of disability in young adults in industrialized societies [157]. The major disabling aspect of MS is the cognitive impairment, which is characterized primarily by memory loss, attention deficits, slowed information processing, and failure in executive function [158].

Some MS symptoms may be closely dependent upon changes in KCN [159]. K_v_1.1 and K_v_1.2 are specifically found in paranodal regions of axons [160]. KCN blockers such as aminopyridines (APs) when applied *in vitro* to demyelinated axons are able to restore the conduction and also to potentiate synaptic transmission [161–164]. Because of that, much attention has been given to K_v_ blockers as a prospective agent for MS and other neuropathies treatments [165, 166].

Moreover, MS is a chronic inflammatory autoimmune disease and the central role of T lymphocytes, and their K_v_1.3 and K_Ca_1.1 channels, in its pathogenesis has been largely evidenced [167–169]. Kaliotoxin, which blocks the lymphocyte K_v_1.3 and the neuronal K_v_1.1 channels, ameliorates the symptoms of adoptive experimental autoimmune encephalomyelitis (EAE) in rats [170], a widely used model for the human MS disease [167]. It is worthy to mention that 4-AP has been used to manage some of the symptoms of MS [171, 172]. 4-AP was approved by the US Food and Drug Administration (FDA) on January 22, 2010 for the treatment of MS [173].

### 5.6. Parkinson's Disease

Parkinson's disease (PD) is a slowly progressive degenerative disorder of the CNS and is characterized by slowness or deficiency of movement (bradykinesia), rigidity, postural instability, and tremor primarily while at rest. The motor symptoms of PD result from the death of dopamine-generating cells in the substantia nigra, a region of the midbrain. In the early stage of the disease, the most obvious symptoms are those that are movement-related, such as shaking, rigidity, slowness of movement, and difficulty with walking and gait. With time, cognitive and behavioral problems may arise, commonly followed by dementia in the advanced phase of the disease. PD is more common in the elderly, with most cases occurring after the age of 50 (for review see [174, 175]).

Potassium channels have been implicated in the pathogenesis of PD. K_ATP_ channels comprised of K_ir_6.2 and Sur1 subunit are abundantly expressed in dopaminergic neurons of substantia nigra [176]. K_ATP_ directly couple the cellular metabolic state to membrane excitability. It has been shown that activity of the mitochondrial respiratory chain complex I (CXI, also known as NADH: ubiquinone reductase) is reduced in PD patients, strongly suggesting that metabolic stress is an important trigger factor for the PD neurodegeneration. Dopaminergic midbrain neurons express different types of K_ATP_ channels mediating their differential response to the inhibition of mitochondrial complex I [177]. The use of CXI inhibitor leads to the activation of SUR1/K_ir_6.2-containing K_ATP_ channels, which results in a membrane hyperpolarization of the dopaminergic neuron that is associated with a complete loss of spontaneous activity [178]. Studies of dopaminergic midbrain neurons in the *weaver* mouse, a genetic mouse model of dopaminergic degeneration similar to that in PD [179], sustain the proposal of K_ATP_ channel activation as a neuroprotective strategy. Up to now, no K_ATP_ channel activators have been described from arthropod venom.

### 5.7. Epilepsy

The term epilepsy refers to a group of neurological disorders, clinically diverse and with multiple etiologies, characterized by paroxysmal brain discharges referred to as spontaneous recurrent seizures [180, 181]. Epilepsy is the second most common neurological disorder (after stroke), affecting 1-2% of the world's population [182]. Failure to treat epilepsy causes neurobiological, psychological, and social consequences for the patient [183], particularly cognitive impairment [184, 185]. Age of onset of epilepsy, type and duration of seizures, as well as the antiepileptic drug, therapy are strongly related to cognitive dysfunction present in the syndrome [186–188].

Clearly, ion channels are critical for regulating excitability and, contribute significantly to epilepsy pathophysiology. In a recent article, N'Gouemo [189] related that the loss-of-function large conductance K_Ca_ channels mutations contribute to neuronal hyperexcitability that can lead to temporal lobe epilepsy, tonic-clonic seizures and alcohol withdrawal seizures, and blockage of these channels can trigger seizures and status epilepticus.

Despite its availability, conventional and “new generation” antiepileptic drugs (AEDs) are commonly associated with side effects, which can vary in frequency and severity [190]. In addition, AEDs are ineffective in controlling seizures in one-third of patients (drug-resistant), reaching 70% in patients with temporal lobe epilepsy, which is the more frequent epilepsy in adults and it is characterized by degeneration of limbic structures (sclerotic hippocampus), directly involved in different memory processes and in their modulation [191–194]. It is noteworthy that cognitive deficits represent a serious neuropsychological problem in people suffering from temporal lobe epilepsy.

Several studies suggest that KCN may be an important new class of targets for anticonvulsant therapies [195, 196], especially for refractory epilepsy. New drugs that act on potassium channels have been provided and approved by the FDA and the European Union, for example, ezogabine (retigabine). *In vitro* studies indicate that ezogabine acts primarily by opening neuronal K_v_7.2–7.5 channels [197].

## 6. Arthropod Toxins: Therapeutic and Study Tools

Given that the dysfunction of K^+^ channels has significant contribution in cognitive deficits of several neurological clinical conditions, the drugs that modulate their activity became important allies in the study and treatment of these pathologies. Due to the wide presence and diversity of potassium channels in the brain, these therapeutic agents need to be specific and potent. In this way, the toxins from arthropods turn out to be excellent candidates.

### 6.1. Potential of KTxs as Pharmacological Tools

KTxs that act on KCN responsible for *I*_(A)_ currents are of particular interest. These channels regulate firing frequency, spike initiation and waveform in excitable cells and may contribute to specialized functions, such as learning, memory, and behavior [146, 147]. According to the cell type and to the molecular heterogeneity, *I*_(A)_ currents exhibit a wide diversity of physiological properties. KCNs of subtype K_v_4 participate in the generation of *I*_(A)_ currents in the cerebellum granular cells [198], neostriatal cholinergic interneurons [199], and hippocampal interneurons [147]. K_v_2 channels seem to be equally important for the existence of *I*_(A)_ currents in neocortical pyramidal neurons [200, 201]. Therefore KTxs that influence the *I*_(A)_ currents, or more specifically in K_v_2 and/or K_v_4 channels activity, become potential pharmacological tools for studying the participation of KCN in learning memory and its dysfunctions.

Many scorpion KTxs influence the *I*_(A)_ currents. Noxiustoxin (NTX), from the venom of the Mexican scorpion *Centruroides noxius* Hoffmann [202], and discrepin, toxin from the Venezuelan scorpion *Tityus discrepans* [203], belong to *α*-KTx15 subfamily. Toxins of this subfamily are reported to affect the *I*_(A)_ currents [198]. Interesting, discrepin blocks irreversible K^+^-channels of rat cerebellum neurons [203]. BmTX3 (systematic name *α*-KTx 15.2) is toxin isolated from venom of Manchurian scorpion *Mesobuthus martensi *is Karsch. This peptide blocked (Kd = 54 nM) completely the *I*_(A)_ current of striatum neurons in culture, whereas the sustained K^+^ current was unaffected. The labeled synthetic toxin (^125^I-sBmTX3) was found in the striatum, hippocampus, superior colliculus, and cerebellum in the adult rat brain [204]. BmTx3B (Martentoxin, systematic name alpha-KTx 16.2) is another toxin isolated from *Mesobuthus martensi* Karsch. It was tested on two types of voltage-dependent potassium currents recorded from dissociated hippocampal neurons of neonatal rat in whole-cell voltage-clamp mode. BmTx3B selectively inhibited the delayed rectifier potassium current (*I*_(*K*)_), without affecting the *I*_(A)_ current [205]. Therefore these scorpions KTxs are candidates to become tools for the study of KCN in physiological and pathological mechanisms of learning and memory.

As mentioned above, the spider venom is a source of KTxs that act on K_v_2 and K_v_4 channels. The first spider toxins described as KCN blockers were the hanatoxins 1 and 2 (HaTx 1 and 2) of the theraphosidae spider *Grammostola spatulata* [206]. Despite the marked differences in their primary sequence, both blocked the K_v_2.1 channel with Kd of 42 nM. HaTxs block K_v_1 and K_v_3 channels, while the *shal*-related channel (type K_v_4) is sensitive to the toxin. The heteropodatoxins (HpTx 1–3), isolated from the venom of *Heteropoda venatoria* (Sparassidae), blocked the conductance of K_v_4.2 but not of K_v_1.4, in a voltage-dependent manner. Other toxins that act on KCN were isolated from the venom of the Theraphosidae *Phrixotrichus auratus*. The phrixotoxins (Patx 1 and 2) specifically block K_v_4.3 and K_v_4.2 currents at nanomolar concentrations altering the gating properties of these channels by interacting with the voltage sensor. The subfamilies of s*haker* (K_v_1), *shab* (K_v_2), and *shaw* (K_v_3) were not inhibited by PaTxs [207]. The stromatoxin1, ScTx1, isolated from tarantula *Stromatopelma calceata*, was the first high-affinity inhibitor described for the K_v_2.2 channel. ScTx1 also inhibited K_v_2.1 channels, K_v_4.2 and K_v_2.1/K_v_9.3 heterodimer [208]. HmTx 1 and 2, peptides purified from the tarantula spider *Heteroscodra maculata*, inhibited potassium currents associated with subtypes K_v_2 [206]. Guangitoxin-1E (GxTX-1E) is a potent gating modifier peptide of K_v_2 channels isolated from the venom of the tarantula *Plesiophrictus guangxiensis *[209]. Other spider toxins targeting the K^+^-channel voltage sensor include heteroscodratoxins (HmTx1,2), which target K_v_2 and K_v_4 channels [206]; TLTx1–3, which preferentially inhibit K_v_4 channels [210]; PhTx3-1, which inhibits the outward rectifier A-type K^+^-channel [211]. SGTx1 (Kappa-theraphotoxin-Scg1a) is a peptide toxin isolated from the venom of the aggressive African Theraphosidae *Scodra griseipes* that has been shown to inhibit outward K^+^ currents in rat cerebellar granule neurons [64]. Functionally, SGTx1 reversibly inhibits potassium currents in oocytes expressing K_v_2.1 channels and acts by shifting the activation of the channel to more depolarized voltages [212]. Finally, phrixotoxin-1, a peptide purified from the venom of the tarantula *Phrixotrichus auratus,* is a specific and potent blocker of K_v_4.3 [207]. So there are many spider KTxs that are potential tools for the study of KCN in learning and memory and its dysfunction.

Toxins that act on large-conductance Ca^2+^-activated K^+^-channels (K_Ca_1.1 or BK) and small-conductance Ca^2+^-activated K^+^ channel (K_Ca_2.1 or SK1, K_Ca_2.2 or SK2, and K_Ca_2.3 or SK3) are important too. Neuronal firing is also regulated by K_Ca_1.1 and K_Ca_2 which constitute an exclusive family of ion channels which combine intracellular chemical changes and electric signaling [213, 214]. K_Ca_1.1 are homologous to K_v_ channel *α*-subunits, but possess additional hydrophobic segments forming an extracellular N-terminal and a long intracellular C-terminal that holds one of the Ca^2+^-binding sites [215]. It has been reported that K_Ca_ channels are involved in regulation of neocortex pyramidal cell excitability [216, 217]. Therefore KTxs that influence the K_Ca_ channels become also potential pharmacological tools.

K_Ca_ channels are blocked by many scorpion toxins. Martentoxin, purified from *Mesobuthus martensi* Karsch venom, is able to block K_Ca_1.1 currents in rat hippocampal neurons [218]. This KCN is also inhibited by slotoxin from *Centruroides noxius* [219] and noxiustoxin from *Centruroides noxius* [220]. Slotoxin is described not only as a potent and selective blocker, but it also can differentially inhibit K_Ca_1.1 channels, depending on the presence of *β*-subunits [219] and on the *α*-splice variant [221]. K_Ca_2 channels are also blocked by different scorpion toxins. For example, there are scyllatoxin (leiurotoxin I) isolated from *Leiurus quinquestriatus hebraeus* [222] and tamapin from *Mesobuthus tumulus* [223]. This last toxin blocks K_Ca_2 channels in pyramidal neurons of the hippocampus as well as in cell lines expressing distinct K_Ca_2 channel subunits, displaying a remarkable selectivity for K_Ca_2.2 (IC50 = 24pM) versus K_Ca_2.1 (*≅*1750-fold) and K_Ca_2.3 (*≅*70-fold) channels [223]. These data reinforce the potential of scorpion KTxs as pharmacological tools.

As has been evident, multiple KCNs participate in the regulation of cellular events behind the phenomena of learning and memory and their dysfunctions. Therefore, it is necessary a varied arsenal of pharmacological tools to investigate the role of each potassium channel in these cognitive processes. It also became clear that scorpions and spiders KTxs can provide this diversity of tools.

## 7. Conclusion

The KCNs are components of the mechanisms responsible for learning and memory. Its diversity and wide distribution in the brain make the action of KCN during the formation of learning and memory retrieval to be complex and variable. To investigate the role of each subtype of KCN in these phenomena, we need also a wide variety of pharmacological tools, specific and potent for KCN. The toxins from the venoms of arthropods can be such tools. These precise potassium channel toxins may not only have an important contribution to uncover the processes underlying learning and memory, but can also become therapeutic agents for many diseases and disorders of the CNS.

## Figures and Tables

**Table 1:** Distribution of different types of K^+^ channels in hippocampus.

Channels	Hippocampus			Employed technique	Ref.
	CA1	CA3	DG		
K_v_1.1	++	+++	+++	ISH, IMH, IMC, and CIMP in hippocampus or brain of rat, mouse, or gerbil.	[37–39]
K_v_1.2	+	+	++		
K_v_1.3	−	−	−	IMH in gerbil hippocampus.	[39]
K_v_1.4	++	++	++	ISH, IMH, and IMC in hippocampus or brain of rat, mouse or gerbil.	[20, 38, 39]
K_v_1.5	+	+	−	IMH or single-cell RT-PCR in gerbil or rat hippocampus.	[39, 40]
K_v_1.6	++	+++	+++	IMH in gerbil hippocampus.	[39]
K_v_2.1	++	++		Single-cell RT-PCR in rat hippocampus.	[17]
K_v_3.1	++	+++	+++	Northern blot analysis and ISH in rat brain.	
K_v_3.2	+++	++	−		[17]
K_v_3.3	+	+	++		
K_v_3.4	−	−	++		
K_v_4.1	+	+	++	ISH in rat brain.	[18]
K_v_4.2	+++	++	+++	ISH or IMH in rat or mouse brain.	[18, 41, 42]
K_v_4.3	+	++	+++	ISH in rat brain.	[18, 42]
K_v_7.2	+++	+++	+++	ISH and IMH in rat brain	
K_v_7.3	+++	+++	+++		
K_v_10.1	++	+++	++	ISH, real time PCR, or IMH in rat brain.	[19, 43]
K_v_10.2	−	−	−	ISH and IMH in rat brain.	
K_v_11.1	++	−	−		
K_v_11.2	−	−	−		[19]
K_v_11.3	+++	−	−		
K_v_12.1	+	−	+		
K_v_12.2	++	−	++		
K_Ca_1.1	+++	+++	+++	ISH, WB analysis, IMH, IMF, IMC, or RLB in mouse or rat brain.	[21, 22, 44]
K_Ca_2.1	++	+++	++	ISH, IB analysis, IMH, or RLB in rat brain.	[23, 24]
K_Ca_2.2	+++	+++	+	ISH, IB analysis, IMH, or RLB in rat brain.	[23–25]
K_Ca_2.3	+	++	+		
K_2P_1.1	−	++	++	ISH in rat and mouse brain.	
K_2P_2.1	++	+	+++	ISH, WB analysis, IMH, IMF, or IMC in rat or mouse brain.	[31]
K_2P_3.1	++	++	++	ISH in rat and mouse brain.	
K_2P_4.1	++	+++	+		[31]
K_2P_9.1	++	++	+++		
K_2P_10.1	−	++	+		
K_ir_2.1	+	+	+++	ISH or IMH in mouse or rat brain.	[26–28]
K_ir_2.2	++	+	++	ISH in mouse or rat brain.	[26, 27]
K_ir_2.3	++	+	+++		
K_ir_3.1	+++	++	+++	ISH or IMH in rat brain.	[27, 28]
K_ir_3.2	+++	+++	+++	ISH or IMH in rat brain.	[27, 45]
K_ir_3.3	+++	+++	+++	ISH in rat brain	[27]
K_ir_3.4	+	++	+	WB analysis, IMH or ISH in rat or mouse brain.	[27, 45–47]
K_ir_6.2	++	++	++	ISH, IMH, or IMF in rat or mouse brain.	[29, 48, 49]

The symbols indicate signal intensity as follows: − (not detected); + (weak); ++ (moderate); +++ (high); CA1, CA3, and DG (Dentate gyrus) are regions of hippocampal formation; Ref.: reference; RT-PCR means reverse transcription polymerase chain reaction; ISH is used for *in situ* hybridization; IMH for immunohistochemistry; IMC for immunocytochemistry; CIMP for coimmunoprecipitation; IMF for immunofluorescence; WB for western blot; IB for immune blot; RLB for radioligand binding.

**Table 2:** K^+^ channels manipulations and their effects on experimental behavioral models for learning and memory.

Channels	Technique	Effect on channel	Behavioral test	Result	Ref.
K_v_, K_Ca_, and K_ir_	icv of minoxidil, pinacidil, TEA, glibenclamine, gliquidone, and cromakalim.	TEA blocks Kv and K_Ca_. Gliquidone and glibenclamide block K_ir_. Minoxidil, pinacidil and cromakalim open K_ir_	PAT in mice	Minoxidil, pinacidil and cromakalim: (−) TEA, glibenclamine, gliquidone: (+)	[50]

K_v_1.1	icv of antisense oligodeoxyribonucleotide to K_v_1.1 mRNA.	Inhibition of channel expression	PAT in mice	(−)	[51]
			MWM in rats	(−)	

K_v_12.2	K_v_12.2 (BEC1) knockout mice	Inhibition of channel expression	MWM	(+)	
			Y-maze test	(+)	
			WFT	(+)	
	K_v_12.2 OVER mice	Overexpression of channel in the forebrain	MWM	(−)	[52]
			Y-maze test	(−)	
			WFT	(−)	

K_Ca_2	Systemic Infusion of EBIO or CyPPA.	EBIO activates SK channels. CyPPA activates K_Ca_2.2 (SK2)/ K_Ca_2.3 (SK3) subunits over K_Ca_2.1 (SK1), and is more potent than EBIO.	ORT in mice	EBIO: (−) CyPPA: (−)	
			Contextual FCP in mice	EBIO: (0) CyPPA: (NT)	[53]
			Tone FCP in mice	EBIO: (0) CyPPA: (NT)	

K_Ca_1.1	ih (CA1) of paxilline	Paxilline blocks the channel.	Trace eyeblink in rats	(−)	[54]

K_Ca_2.2	SK2-OVER mice	Overexpression of K_Ca_2.2 (SK2) protein and K_Ca_2.2 mRNA	MWM	(−)	
			Contextual FCP	(−)	[55]
			Tone FCP	(−)	

K_Ca_2.2	SK2-OVER mice	Overexpression of K_Ca_2.2 (SK2) protein and K_Ca_2.2 mRNA	Contextual FCP	(−)	[56]

K_Ca_2.3	Doxycycline-induced conditional SK3 channel deficient (T/T) mice	Inhibition of channel expression	PAT	(0)	
			MWM	(0)	
			ORT	(0)	
			Y-maze test	(−)	[57]
			Five-trial inhibitory avoidance test	(−)	

K_2P_10.1	Infusion of siRNA in the EC.	Knock down K_2p_10.1 channels in the EC.	MWM in rats	(+)	[58]

K_ir_3.4	K_ir_3.4 (GIRK4) knockout mice	Inhibition of channel expression	PAT	(0)	[51]
			MWM	(−)	

K_ir_6.2	ih (CA3) of diazoxide, or tolbutamide, or both.	Diazoxide opens the channel. Tolbutamide blocks the channel.	Contextual FCP in mice	Diazoxide: (−) Tolbutamide: (0) Both: (0)	[48]
			Tone FCP in mice	Diazoxide, tolbutamide or both: (0)	
	K_ir_6.2 knockout mice	Inhibition of channel expression	Contextual FCP	(−)	
			Tone FCP	(−)	
			MWM	(0/−)	

(−): impairment; (+): improved; (0): neutral; (0/−): slight impairment; (NT): not tested; Ref.: reference; icv: intracerebroventricular injection; ih: intra-hippocampal injection; EC: entorhinal cortex; OVER: overexpressing; EBIO: 1-ethyl-2-benzimidazolinone; CyPPA: Cyclohexyl-[2-(3,5-dimethyl-pyrazol-1-yl)-6-methyl-pyrimidin-4-yl]-amine; siRNA: small interfering RNA; PAT is used for passive-avoidance test; MWM for Morris water maze test; WFT for Water-finding task; ORT for object recognition task; FCP for fear-conditioning paradigm.

**Table 3:** Assembled data on the effect of the bee and scorpion venom KTxs on the performance of animals in behavioral tests of learning and memory.

	Species	Toxin	KCN target	Behavioral test	Result	Ref.
Scorpion	*Androctonus mauretanicus mauretanicus*	Kaliotoxin	K_v_1.1 and K_v_1.3 blocker	Olfactory discrimination task in rats	Improvement	[92]
	*Buthus tasmulus*	Iberiotoxin (IbTx)	K_Ca_1.1 blocker	Passive avoidance test in chicks	Impaired retention	[93]
	*Leiurus quinquestriatus hebraeus *	Charybdotoxin	K_v_1.3 and K_Ca_1.1 blocker	Passive avoidance test in mice	Improvement	[50]
		Lei-Dab7	K_Ca_2.2 blocker	Radial arm maze in rat	No effect	[25]

Bee	*Apis mellifera*	Apamin	K_Ca_2.2 and K_Ca_2.3 blocker	Bar-pressing response in appetitively motivated mice	Improvement	[94]
				Object recognition task in rats	Improvement	[95]
				Habituation task in rats	Improvement	[96]
				Passive avoidance test in rats or mice	No effect	[96, 97]
				Passive avoidance test in mice	Improvement	[50, 98]
				Morris water maze in mice	Improvement	[97, 98]
				Morris water maze in rats	No effect	[98]
				Y-maze test in mice	No effect	[97]
				Radial arm maze test in mice or rats	Improvement	[25, 99]
				Visual discrimination in rats	No effect	[98]
				Olfactory discrimination task in rats	Improvement	[100]
				Olfactory associative task in rats	Improvement	[101]
				Tone fear-conditioning paradigm in mice	No effect	[53]
				T-maze test in rats	Improvement	[102]
				Passive avoidance task in chicks	Impaired retention	[103]

Ref.: reference.
